# Efficacy evaluation of ethanolic extract of *Tamarindus indica* L. leaves as possible alternate therapy in septic arthritis model of rabbit

**DOI:** 10.1186/s12906-019-2676-4

**Published:** 2019-09-18

**Authors:** Bishnu Prasad Sinha, Souvick Chatterjee, Rinku Buragohain, Indranil Samanta, Siddhartha Narayan Joardar, Prasenjit Mukherjee, Asit Kumar Maji, Partha Das, Tapan Kumar Mandal, Tapas Kumar Sar

**Affiliations:** 1 Department of Veterinary Pharmacology & Toxicology, West Bengal University of Animal & Fishery Sciences, 37 Khudiram Bose Sarani, Kolkata, West Bengal 700037 India; 2 Department of Veterinary Microbiology, West Bengal University of Animal & Fishery Sciences, 37 Khudiram Bose Sarani, Kolkata, West Bengal 700037 India; 3 Department of Veterinary Surgery & Radiology, West Bengal University of Animal & Fishery Sciences, 37 Khudiram Bose Sarani, Kolkata, West Bengal 700037 India; 4 Department of Veterinary Anatomy & Histology, West Bengal University of Animal & Fishery Sciences, 37 Khudiram Bose Sarani, Kolkata, West Bengal 700037 India

**Keywords:** Anti-microbial activity, Ethanolic extract, Septic arthritis, *Staphylococcus aureus*, *Tamarindus indica* L. leaves

## Abstract

**Background:**

Our previous study exhibited free radicals scavenging and antioxidant activities of ethanolic and aqueous extracts of *Tamarindus indica* L. leaves in chronic sodium fluoride poisoning in rats. *Tamarindus indica* L. seed extract was also reported to have anti-arthritic efficacy by inhibiting cartilage and bone degrading factors. Therefore, an attempt was made to evaluate the effects of ethanolic extract of *Tamarindus indica* L. leaves in septic arthritis.

**Methods:**

The safety study was performed by oral dosing of ethanolic extract of the plant leaves at 2 g kg^− 1^ for consecutive 28 days in rabbits. Septic arthritis was induced in rabbits by single intra-articular inoculation of 10^4^ c.f.u. of *Staphylococcus aureus* to the left stifle joint and was monitored by bacterial colony count, some relevant biochemical parameters and histopathological interpretation of the affected joint. For efficacy evaluation in septic arthritis, linezolid at 75 mg kg^− 1^ twice daily for 10 days and the ethanolic extract of *Tamarindus indica* L. at 500 and 1000 mg kg^− 1^ for consecutive 14 days were administered orally to the rabbits after 48 h of induction of arthritis.

**Results:**

In sub-acute toxicity study of *Tamarindus indica* L. leaves ethanolic extract, no significant change between days was found for aspertate aminotransferase, alanine transaminase, alkaline phosphatase, blood urea nitrogen and creatinine compared to day 0 values of the same group. The bacterial colony count of synovial fluid following *Staphylococcus aureus* inoculation to left stifle joint was found to be 1.08 ± 0.47 and 1.19 ± 0.29 c.f.u. mL^− 1^ in ethanolic extract low dose and high dose groups respectively, on day 2 which was reduced to 0.057 ± 0.036 c.f.u. mL^− 1^ and nil on day 16. The test extract was also found to markedly reduce simultaneous glucose difference, total protein ratio of serum and synovial fluid, joint radius and joint narrowing.

**Conclusion:**

Ethanolic extract of *Tamarindus indica* L. leaves at 500 mg kg^− 1^ and 1000 mg kg^− 1^ produced anti-arthritic effects against *S. aureus* induced septic arthritis in rabbits. However, the ethanolic extract at 1000 mg kg^− 1^ orally for consecutive 14 days showed better effects in septic arthritis.

## Background

Septic arthritis is inflammation of joint caused by bacterial infection. The inflammatory process due to bacterial infection results in local join destruction. The joint infection may also be accompanied with systemic infection that may lead to morbidity and mortality [1]. The rigorous advancement in arthritis research has endorsed the use of medicinal plants in septic arthritis treatment. The plant part like *Tamarindus indica* L. leaves containing good quantity of vitamin C, α-carotene, minerals like phosphorus, potassium, calcium, magnesium and antimicrobial properties have the potential to be used as possible alternate therapy in septic arthritis. *Tamarindus indica* L. leaves extract was reported to have antimicrobial activities against both gram negative and gram positive bacteria [2, 3]. The most pronounced antimicrobial activity was produced by ethanolic extract and the highest minimum inhibitory concentration (MIC) and minimum bactericidal concentration (MBC) were exhibited against *Pseudomonas aeruginosa* and methicillin resistant *Staphylococcus aureus* (MRSA) [2]. The ethanolic extract of *Tamarindus indica* L. leaves at 400 mg kg^− 1^ was reported to produce analgesic activity comparable to 25 mg kg^− 1^ dose of diclofenac sodium in mice [4]. In addition, the hydroethanolic extract of *Tamarindus indica* L. leaves was reported to have potential anti-inflammatory as well as anti-nociceptive actions in paw oedema induced by carrageenan in rats [5]. The ethanolic and aqueous extracts of *Tamarindus indica* L. seed coat were examined for anti-arthritic activity against Freund’s complete adjuvant induced paw oedema and arthritis in Wister albino rats of either sex. Both the extracts were found to significantly inhibit paw oedema induced arthritis which was evidenced by reduced interleukin (IL) expression and prostaglandin E_2_ (PGE_2_) production. The results supported anti-inflammatory, anti-nociceptive and anti-arthritic effects of *Tamarindus indica* L. in arthritis animal model [6]. Ethanolic extract of *Tamarindus indica* L. seed was proposed to block over production of pro-inflammatory mediators maintaining body antioxidant system homeostasis in arthritis. The extract was recorded to produce anti-arthritic effect by inhibiting both enzymatic and non-enzymatic factors responsible for bone and cartilage degradation [7]. Another study was conducted to evaluate the effect of tamarind fruit paste on the rate of wound healing in rabbits. It was reported that wounds treated with tamarind show a faster rate of wound closure compared to the control group [8]. The methanolic extract of *Tamarindus indica* L. leaves, stem bark, seeds, fruit pulp, fruit bark and roots were examined for hepatoprotective and nephroprotective effect in acute and chronic carbon tetrachloride induced organ injuries in rats. Bilirubin, serum glutamic oxaloacetic transaminase (SGOT) and serum glutamic pyruvic transaminase (SGPT) were evaluated as liver markers whereas urea and creatinine were monitored for renal failure. Extract treatment caused a significant decrease in the activities of SGOT, SGPT, bilirubin, urea and creatinine in induced hepatopathic and nephropathic rats [9]. Acute toxicity study of ethanolic extract of *Tamarindus indica* L. (EETI) seed at 2000 mg kg^− 1^ oral dose was also reported to produce neither any adverse effect nor mortality till 14 days in Wistar albino rats [10]. The phytochemical study of *Tamarindus indica* L. leaves extract was reported to contain tannins, saponins, sesquiterpenes, alkaloids and flavone glycosides like orientin and vitexin [11]. Our previous study also reported free radicals scavenging and antioxidant activities of both ethanolic and aqueous extracts of *Tamarindus indica* L. leaves in chronic sodium fluoride induced oxidative stress in rats [12]. But, no infectious disease model study has been performed yet to check the efficacy of *Tamarindus indica* L. leaves extract against sepsis and inflammation together in a living system particularly in septic arthritis. Several studies reported better efficacy of combined therapy of antibiotic and corticosteroid compared to only antibiotic therapy in treatment of septic arthritis in different animal species [13, 14]. Therefore, a comparative study was conducted between oral therapy of ethanolic extract of *Tamarindus indica* L. leaves and linezolid (LNZ) in rabbits as LNZ was found to be one of the most effective antibiotics against *Staphylococcus aureus* (including MRSA) infection particularly in septic arthritis [15]. Septic arthritis caused by *S. aureus* infection or gram negative bacteria requires 4 weeks of parenteral antibacterial drug therapy in humans which is quite long period [16, 17]. Development of herbal therapy as an alternate can minimize both cost and adverse effects of long term antibiotic treatment. The safety study of ethanolic extract of *Tamarindus indica* L. leaves in septic arthritis showed the extract as practically non-toxic in the present study which was another advantage for the extract as an alternate therapy for septic arthritis. Therefore, the present study was aimed to evaluate and compare the efficacy of linezolid alone and ethanolic extract of *Tamarindus indica* L. leaves for treatment of septic arthritis by evaluating bacteriological, biochemical, radiological and histomorphological parameters towards development of a safer and cost effective therapy for septic arthritis.

## Methods

### Animals

Clinically healthy New Zealand white rabbits (*Oryctolagus cuniculus*) of 6 to 8 months of age, weighing 2–2.5 kg were housed individually in custom-made stainless steel metabolic cages and were provided standard feed. The animals were obtained from M/S Chakraborty Enterprise, 3/1D, Girish Vidyaratna Lane, Narkeldanga, Kolkata-700,011, West Bengal, India (Registration number – 1443/PO/b/11/CPCSEA). Animals were maintained in controlled environment with artificial lighting facilities where room temperature was maintained at 26 ± 3 °C. Management and care of the animals were carried out by the veterinarians.

### Plant material

The leaves were collected from *Tamarindus indica* L. in the month of December from the university campus, West Bengal University of Animal and Fishery sciences, Mohanpur, Nadia. The plant material was identified by Dr. Rabindranath Sar (PhD in Botany, Calcutta University) which was authenticated by a botanist at Botanical Survey of India, West Bengal, India (Specimen no.- WBUAFS/LJ 03). A voucher specimen was deposited to Botanical Survey of India, West Bengal, India and another voucher specimen was kept in the Department of Veterinary Pharmacology and Toxicology, West Bengal University of Animal and Fishery Sciences, India. Dust free clean leaves were dried in shade and powdered using a mechanical grinder and subsequently stored in airtight containers.

### Drugs and chemicals

LNZ tablets were obtained from Lupin Pharmaceuticals Private Limited. (Mumbai, India). Other chemicals and kits used in this study were obtained from Promega (USA), Rankem (India), E. Merck (India) and Sigma-Aldrich (Saint Louis, Missouri).

### Ethanolic extraction of *Tamarindus indica* L. leaves

Ethanolic extraction of *Tamarindus indica* L. leave powder was done using Soxhlet apparatus as per reported method [18]. Ethanol was removed from the extract by drying at 45 °C in vacuum evaporator to determine the concentration (mg mL^− 1^) and the final concentrated extract was stored at 4 °C until use in sterile airtight containers.

### Safety study

#### Acute oral toxicity study

Acute oral toxicity study for the test extract was conducted as per OECD (Organisation for Economic Co-operation and Development) guidelines 423. The test dose 5000 mg kg^− 1^ was administered orally to an individual rat. As mortality was not observed, additional two animals of same sex and another three of another sex were again dosed at 5000 mg kg^− 1^. They were monitored for any adverse clinical sign and mortality up to 48 h.

#### Sub-acute oral toxicity study

A total of 12 rabbits were divided randomly into 2 groups each containing 6 animals. Ethanolic extract of *Tamarindus indica* L. leaves (EETIL) at 2 g kg^− 1^ body weight and 2% tween 20 in distilled water were orally administered for 28 consecutive days to two groups, respectively. Biochemical parameters like aspartate aminotransferase (AST) and alanine transaminase (ALT) level, alkaline phosphatase (ALP) activity, blood urea nitrogen (BUN) and creatinine were monitored on different days.

#### Isolation and identification of bacteria

The synovial fluid and exudate from lesion of a three months old black Bengal kid suffering from septic arthritis was collected aseptically in sterile vial. The clinical sample was inoculated into nutrient broth (HiMedia, India) and incubated at 37 °C for overnight. The growth on the next day was transferred into mannitol salt agar (HiMedia, India) and incubated at 37 °C for 24 h. Characteristic colonies surrounded by bright yellow zone were selected for confirmation. The selected single colony was transferred into nutrient agar slant. The colonies were preliminarily identified by Gram’s staining, standard biochemical tests such as catalase, oxidase, urease, carbohydrate fermentation with glucose, sucrose, maltose, mannitol [19].

#### PCR based confirmation of *Staphylococcus aureus* isolates

*S. aureus* isolates identification based on biochemical tests were confirmed by possession of *nuc* gene in PCR (polymerase chain reaction). The primers and cycle condition for *nuc* detection, PCR was adopted from earlier work [20].

#### Detection of antibiotic resistance of *Staphylococcus aureus* isolates

The confirmed *S. aureus* isolates were subjected to antibiotic sensitivity test with linezolid (Bio-Rad), methicillin, ampicillin, ampicillin-sulbactum, amoxicillin-clavulanic acid, ticarcillin-clavulanic acid, imipenem-ethylenediaminetetraacetic acid, piperacillin-tazobactam, cefotaxime, ceftizoxime, ceftriaxone, ceftriaxone-tazobactam, enrofloxacin, ciprofloxacin, vancomycin and gentamicin antibiotic discs procured from HiMedia, India following CLSI (Clinical & Laboratory Standards Institute) guidelines [21].

#### MIC of linezolid

MIC of linezolid had been performed as per protocol using standard strip [Linezolid EzyMICTM Strip (0.016–256 μg mL^− 1^)] (HiMedia, India).

#### Induction of septic arthritis in rabbits with *Staphylococcus aureus* isolates

The rabbits were inoculated intra-articularly with single dose of 18 h old *S. aureus* culture (1 mL) possessing 10^4^ c.f.u. (colony forming unit) mL^− 1^ concentration in the left stifle joint. Arthritis was confirmed by evaluating clinical symptoms like swelling, redness of the particular joint, lameness, restricted movement, restlessness, anorexia and characteristics of synovial fluid in the inoculated joint.

#### Experimental design

A total of 24 rabbits were divided randomly into 4 groups each containing 6 animals. Six apparently healthy female rabbits were utilized for induction of septic arthritis and were considered as negative control (Gr-I). Another six apparently healthy female rabbits (Gr-II) were utilized for induction of septic arthritis and LNZ was administered at 75 mg kg^− 1^ orally twice daily for 10 days which was considered as positive control. In addition, Gr-III containing six apparently healthy female rabbits following induction of septic arthritis was given ethanolic extract of *Tamarindus indica* L. leaves (EETIL) at 500 mg kg^− 1^ (low dose ethanolic extract of *Tamarindus indica* L. leaves: LDEETIL) orally once daily for 14 days. Other six apparently healthy female rabbits in Gr-IV after induction of septic arthritis were administered ethanolic extract of *Tamarindus indica* L. leaves (EETIL) at 1000 mg kg^− 1^ (high dose ethanolic extract of *Tamarindus indica* L. leaves: HDEETIL) orally once daily for 14 days. LNZ and EETIL at low and high dose were employed after 48 h of induction of septic arthritis in Gr-II, Gr-III and Gr-IV, respectively. The day 0 values of different parameters for all the groups without any treatment or inoculation were considered as baseline values. All the groups contained six animals for comparative study and to assess statistical significance.

#### Collection of blood

Blood samples were collected from the marginal vein using tuberculin syringe after proper cleaning of the ear with 95% v/v (volume by volume) alcohol and application of local anaesthetic cream. Gentle pressure was applied using sterile cotton gauge to the collection site to stop the bleeding after blood collection.

#### Arthrocentesis

Knee arthrocentesis of the rabbit was performed via a para-patellar approach. The skin was prepared with sterile solution before collection.

#### Analysis of biochemical parameters

Biochemical parameters were analysed in both serum and synovial fluid samples. Synovial fluid samples were centrifuged at 3500 rpm (revolutions per minute) for 15 min and supernatants were taken for analysis [22]. Total protein, lactate dehydrogenase (LDH) and glucose were measured by using standard kits in a standard semi-automatic biochemical analyser.

#### Bacterial colony count

Bacterial colony count was conducted as per the standard protocol [19].

#### Joint space (lateral and medial) measurement

Lateral and medial joint space width between distal end of femur and proximal end of tibia-fibula were measured by digital radiograph (AGFA CR 15-X, Canada) with the help of a software (AGFA CR NX 2.0, Canada) on different days in each animal of all the groups before and after induction of septic arthritis as well as during and after the treatment. The angle between tibia-fibula and femur were maintained at a more or less similar degree during radiography.

#### Histomorphological analysis

The animals were euthanized during daytime with intravenous administration of ketamine at 150 mg kg^− 1^ (standardized veterinary dose) to reduce pain during slaughter for collection of menesci. The menisci were dissected and fixed in 10% formalin for overnight, and were decalcified in 5% trichloroacetic acid for approximately 8 days. The samples were dehydrated in alcohols 70, 80, and 90% for 1 h each and kept in 95% alcohol overnight. Subsequently the menisci samples were immersed in four containers of cent percent alcohol for 1 h each and then processed for paraffin embedding. Sections of 5 μm were made by Olympus CUT 4055 microtome and these were stained with hematoxylin and eosin. Table 1 shows the parameters on the basis of which the scoring had been done. Histomorphological scoring parameters were done during daytime for clear observations according to Salter et al. (1981) [23].

**Table 1:** Histomorphological scoring parameters (Salter et al. 1981)

Parameters	Scoring
Cellularity of cartilage	
Normal	0
< 10%	1
10–25%	2
>25%	3
Loss of matrix (erosions)	
Normal	0
< 10%	1
10–25%	2
>25%	3
Cloning of chondrocytes	
Normal	0
< 10%	1
10–25%	2
>25%	3
Adhesions (pannus)	
No Adhesions	0
Covering only margin of cartilage	1
Covering < 50%	2
Covering >50%	3
Grey reads (Red value) ^a^ with Safranin-O	
Values more than 160	0
Values within 140–160	1
Values within 120–140	2
Values within 100–120	3
Values within 80–100	4

^a^Grey reads had been taken instead of orthochromasia in Salter’s study

#### Image analysis

The various parameters were scored with the assistance of image analysis software LEICA QWIN under LEICA DM 2000 microscope considering the pre-scheduled gradation. The slides were interpreted in one session and in same lighting condition. Cellularity of cartilages was determined by the presence of nuclei per square area of cartilage matrix. A population that was three standard deviations less than the normal control cartilage was defined as acellular cartilage. The percentage loss of matrix was calculated as length of surface with eroded matrix divided by total surface length in a photo-micrograph. Clustering of chondrocytes was estimated as number of clones formed divided by total number of chondrocytes in a particular photo-micrograph. Pannus formation was evaluated by the ratio of the length of joint surface covered by pannus and total length of joint surface in a photo-micrograph.

Instead of orthochromasia in Salter’s study, proteoglycans (appeared red) optical density was measured over green background from safranin-o stained field. Two controls were considered for interpretation keeping one as normal cartilage (containing a large amount of proteoglycans and showing a higher value of optical density) and another as untreated infection control cartilage (containing very less amount of proteoglycans and showing a lower value of optical density). The values were measured in a scale where maximum red value was 255 and minimum red value was zero.

### Statistical analysis

The data were expressed as mean and standard error. Student’s t-test was performed to compare the data between different days within the same group of animals.

## Results

All six animals in each group were included in the analysis of each parameter.

### Safety study

#### Acute oral toxicity study

Ethanolic extract of *Tamarindus indica* L. leaves did not show any toxic or adverse effect at 5000 mg kg^− 1^ following single oral dosing in rabbit.

#### Sub-acute oral toxicity study

During the sub-acute oral toxicity study, no significant change in AST, ALT, ALP, BUN and creatinine values was recorded on 7th, 14th, 21st and 30th day compared to the values of 0 day of the same group (Table 2). Therefore, two dose rates (500 mg kg^− 1^, 1000 mg kg^− 1^) below 2000 mg kg^− 1^ were selected for efficacy study.

**Table 2:** Mean ± S.E. values of biochemical parameters like ALT, AST, ALP, BUN and creatinine level following oral administration of ethanolic extract of *Tamarindus indica* L. leaves at 2 g kg^− 1^ and 2% Tween 20, at the same dose rate, seperately to two groups of rabbits for consecutive 28 days

Parameters	Groups	Day 0	Day 7	Day 14	Day 21	Day 30
ALT level (IU L^− 1^)	EETIL	43.41^a^ ± 4.22	44.60^a^ ± 5.29	45.00^a^ ± 4.0	43.34^a^ ± 5.7	44.45^a^ ± 3.95
	2% Tween 20	42.21^a^ ± 1.82	43.20^a^ ± 1.79	45.08^a^ ± 1.74	45.80^a^ ± 1.72	44.74^a^ ± 1.63
AST level (IU L^− 1^)	EETIL	43.91^a^ ± 9.78	46.05^a^ ± 8.08	46.62^a^ ± 7.77	46.45^a^ ± 8.73	47.24^a^ ± 11.03
	2% Tween 20	44.21^a^ ± 4.16	46.15^a^ ± 4.45	48.15^a^ ± 3.91	47.14^a^ ± 4.04	46.04^a^ ± 3.80
ALP level (IU L^− 1^)	EETIL	6.98^a^ ± 0.75	7.08^a^ ± 0.33	6.89^a^ ± 0.72	5.90^a^ ± 1.20	6.30^a^ ± 1.28
	2% Tween 20	6.72^a^ ± 0.79	6.88^a^ ± 0.76	7.25^a^ ± 0.81	7.55^a^ ± 0.86	7.25^a^ ± 0.82
BUN level (mg dL^− 1^)	EETIL	16.55^a^ ± 2.17	16.59^a^ ± 1.42	19.72^a^ ± 2.10	20.64^a^ ± 2.41	17.77^a^ ± 1.95
	2% Tween 20	18.19^a^ ± 1.38	18.76^a^ ± 1.18	19.8^a^ ± 1.38	21.31^a^ ± 1.62	21.00^a^ ± 1.21
Creatinine level (mg dL^− 1^)	EETIL	1.48^a^ ± 0.18	1.70^a^ ± 0.17	1.77^a^ ± 0.13	1.83^a^ ± 0.19	1.80^a^ ± 0.15
	2% Tween 20	1.52^a^ ± 0.18	1.56^a^ ± 0.15	1.70^a^ ± 0.17	1.57^a^ ± 0.16	1.50^a^ ± 0.14

Mean bearing a common superscript in a row do not vary significantly (*P* < 0.05) [*n* = 6]

#### Isolation and identification of *Staphylococcus aureus* from goats suffering with arthritis

*Staphylococcus aureus* was isolated and identified by standard biochemical tests from goats suffering with arthritis. *Staphylococcus aureus* isolates from goats suffering with arthritis were detected to be catalase (+ve), oxidase (−ve), urease (+ve) and all the isolates produced acid without gas in glucose, sucrose, maltose and mannitol fermentation which are considered as typical characteristics of standard cultures. All the biochemically confirmed cultures produced ‘nuc’ gene in PCR considered as molecular marker for *S. aureus*.

#### Detection of antibiotic resistance of *Staphylococcus aureus* isolates

*S. aureus* isolates were found resistant against ampicillin, methicillin, cefotaxime, ceftizoxime, gentamicin and ampicillin-sulbactum, amoxicillin-clavulinic acid, ticarcillin-clavulanic acid combinations. However, it was sensitive to linezolid.

#### Confirmation of septic arthritis

Bacterial colony count, LDH level in synovial fluid and gross and histopathological changes along with observation of clinical signs like lameness, swelling and pain sensation at the inoculated joints confirmed septic arthritis in rabbits.

#### Efficacy evaluation

**Fig. 1:**
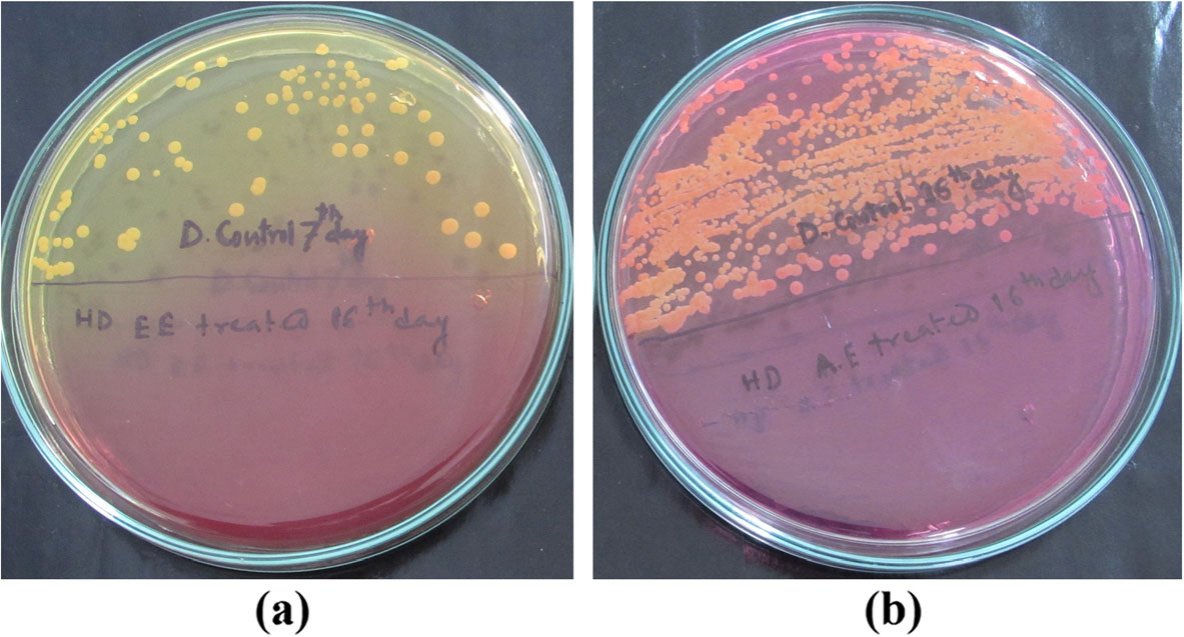
**a** Upper half of the plate showing colonies of *S. aureus* on 7th day post infection in arthritic control group and lower half showing no growth of bacteria on 16th day post infection in HDEETIL treated group. **b** Upper half of the plate showing uncountable colonies of *S.aureus* colonies on day 16th day post infection in arthritic control group

Bacterial colony count showed a significant increase in colony population on day 7 (10.83 ± 3.20 c.f.u. mL^− 1^**)** as compared to day 2 (1.19 ± 0.49 c.f.u. mL^− 1^**)** in arthritic control group (Fig. 1a). But, a significant decrease was recorded on day 7 in LNZ (0.28 ± 0.036 c.f.u. mL^− 1^) and HDEETIL (0.34 ± 0.033 c.f.u. mL^− 1^) treated group as compared to day 2 of the same groups (Table 3). A significant decrease in bacterial population was observed in LDEETIL treated group on day 16 (0.057 ± 0.036 c.f.u. mL^− 1^**)** as compared to day 2 whereas colony population was nil in LNZ and HDEETIL treated groups on day 16 (Fig. 1a). However, colony forming units were plenty in number and very difficult to count in arthritic control group on day 16 (Fig. 1b).

**Table 3:** Number of *Staphylococcus aureus* colonies (c.f.u. mL^− 1^) isolated from synovial fluid of left stifle joint in different groups on different days interval

Groups	Day 2	Day 7	Day 16
Gr-I	1.19^a^ ± 0.49	10.83^b^ ± 3.20	Uncountable
Gr-II	1.11^b^ ± 0.19	0.28^a^ ± 0.036	nil
Gr-III	1.08^c^ ± 0.47	0.79^b^ ± 0.30	0.057 ± 0.036^a^
Gr-IV	1.19^b^ ± 0.29	0.34^a^ ± 0.033	nil

Mean bearing a common superscript (a, b, c) in a row do not vary significantly (*P* < 0.05) [*n* = 6]

**Fig. 2:**
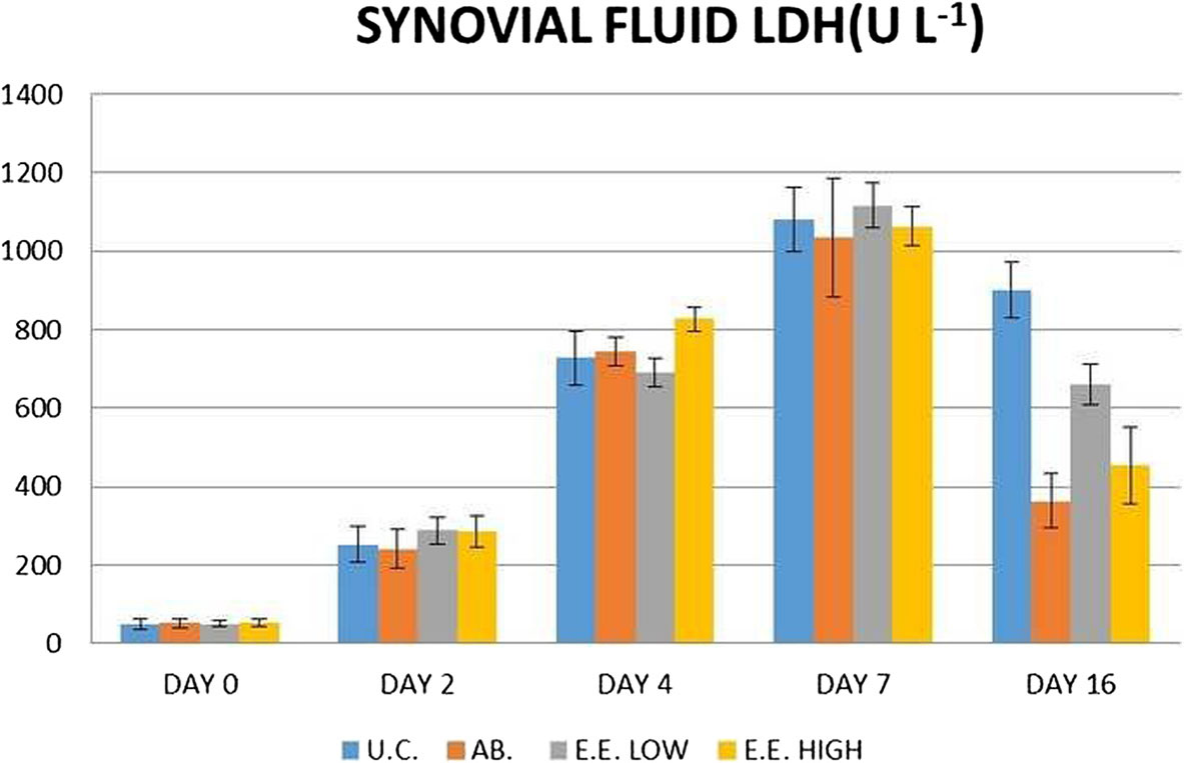
Mean ± S.E. synovial LDH level of different groups on different days

The LDH level of synovial fluid was increased from day 0 to day 7 in all the groups. The LDH level was reduced more markedly in different treatment groups while it was found to be less marked in the arthritic control group on day 16 (Fig. 2 and Table 4).

**Table 4:** Synovial lactate dehydrogenase activity (U L^− 1^) in different groups on different days

Group	Day 0	Day 2	Day 4	Day 7	Day 16
Gr-I	49.40^a^ ± 11.79	252.35^b^ ± 45.59	728.72^c^ ± 68.81	1081.15^d^ ± 81.79	902.09^d^ ± 69.66
Gr-II	51.22^a^ ± 10.28	241.85^b^ ± 48.37	745.40^c^ ± 36.12	1034.14^d^ ± 151.02	363.67^b^ ± 69.68
Gr-III	50.75^a^ ± 8.37	289.58^b^ ± 34.63	691.56^c^ ± 38.13	1116.42^d^ ± 57.93	660.83^c^ ± 50.34
Gr-IV	52.31^a^ ± 8.88	286.60^b^ ± 40.01	828.36^d^ ± 30.76	1064.03^e^ ± 51.02	453.97^c^ ± 97.07

Means bearing a common superscript (a, b, c, d, e) in a row do not vary significantly (*P* < 0.05) [*n* = 6]

The simultaneous difference in glucose level between serum and synovial fluid was observed to be gradually increased up to day 7 in all the groups (> 30 mg dL^− 1^). On day 16, a significant reduction below 25 mg dL^− 1^ was observed in rabbits of Gr-II and Gr-IV but the reduction was non-significant (> 25 mg dL^− 1^) in Gr-I and Gr-III. However in Gr-III, the difference was relatively lower than Gr-I (Table 5).

**Table 5:** Simultaneous difference of serum and synovial fluid glucose level (mg dL^− 1^) in different groups on different days

Groups	Day 0	Day2	Day 4	Day 7	Day 16
Gr-I	13.23^p^ ± 0.97	20.63^p^ ± 3.73	36.36^q^ ± 6.59	47.69^r^ ± 12.11	44.49^qr^ ± 11.64
Gr-II	13.25^p^ ± 2.13	18.24^p^ ± 2.29	30.32^q^ ± 2.40	35.12^q^ ± 3.02	21.60^p^ ± 3.58
Gr-III	13.67^p^ ± 1.06	18.35^p^ ± 1.10	33.96^q^ ± 5.92	39.47^q^ ± 7.45	32.88^q^ ± 6.42
Gr-IV	11.38^p^ ± 0.69	19.22^q^ ± 2.79	30.72^r^ ± 3.65	34.34^r^ ± 2.64	22.51^q^ ± 1.91

Means bearing a common superscript (p, q, r) in a row do not vary significantly (*P* < 0.05) [*n* = 6]

A Significant decline in the simultaneous ratio of total protein between serum and synovial fluid in all the groups were observed at least up to day 4 (≤ 1.25) (Table 6). The ratio in arthritic rabbits approached the normal ratio of healthy rabbits in all the treated groups on day 16 (≥ 2.33). But in arthritic control group, it remained as a value of < 1.

**Table 6:** Simultaneous ratio of total protein in serum and synovial fluid in different groups on different days

Groups	Day 0	Day 2	Day 4	Day 7	Day 16
Gr-I	3.73^q^ ± 0.32	1.56^p^ ± 0.18	1.11^p^ ± 0.14	0.92^p^ ± 0.12	0.94^p^ ± 0.12
Gr-II	3.52^q^ ± 0.34	1.51^p^ ± 0.10	1.15^p^ ± 0.04	1.26^p^ ± 0.14	2.80^q^ ± 0.44
Gr-III	3.83^r^ ± 0.28	1.61^p^ ± 0.17	1.12^p^ ± 0.11	1.15^p^ ± 0.07	2.33^q^ ± 0.18
Gr-IV	3.73^q^ ± 0.10	1.74^p^ ± 0.15	1.25^p^ ± 0.10	1.60^p^ ± 0.21	2.91^q^ ± 0.57

Means bearing a common superscripts (p, q, r) do not differ significantly (*P* < 0.05) [*n* = 6]

**Fig. 3:**
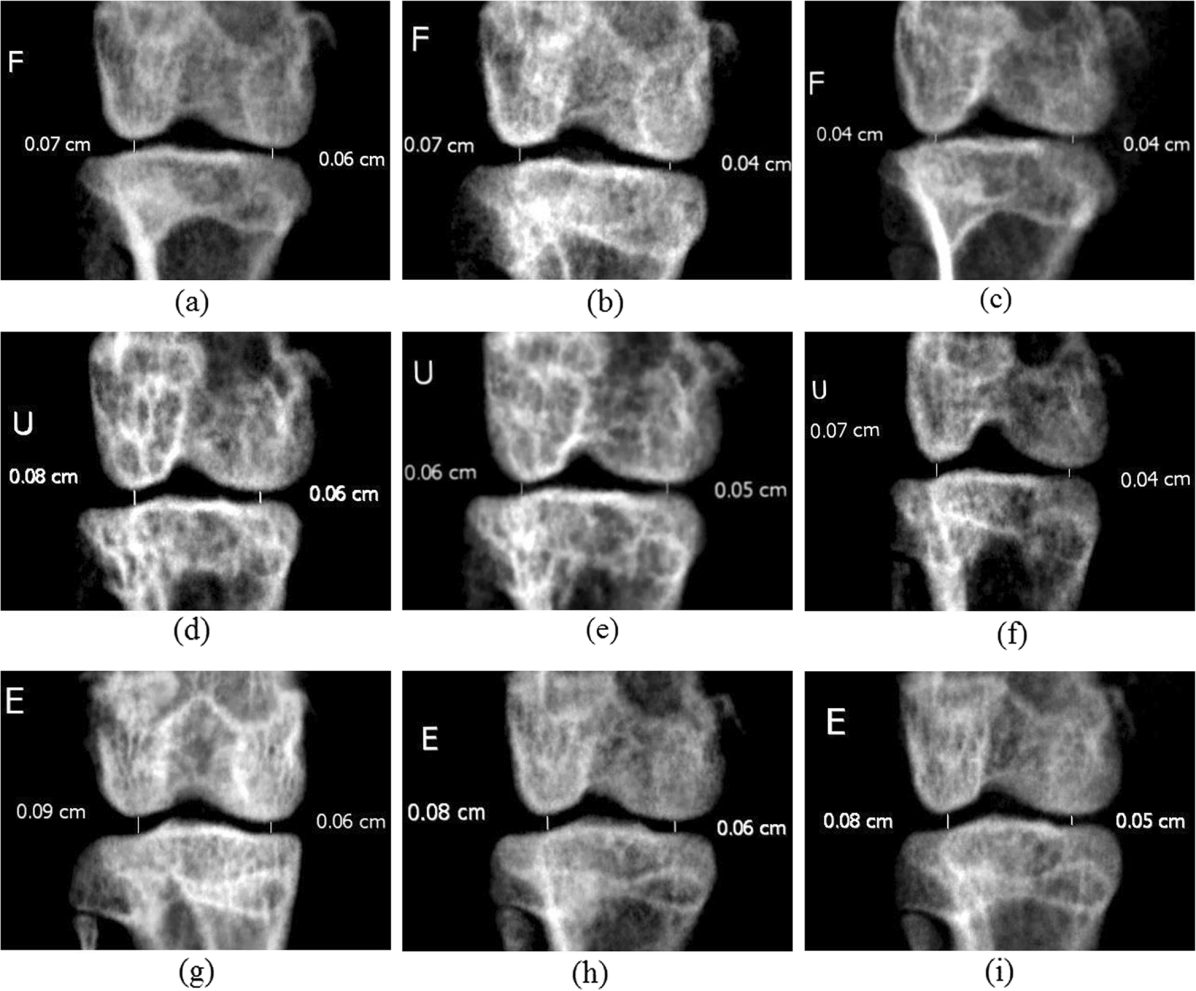
Digital radiographs are showing the stifle joint of untreated arthritic control (**a**, **b**, **c**) rabbits on day 0, 7 and 16, respectively; linezolid treated (**d**, **e**, **f**) rabbits on day 0, 7 and 16, respectively) and HDEETIL treated (**g**, **h**, **i**) rabbit on day 0, 7 and 16, respectively

A marked improvement in joint space (lateral and medial) was noticed on day 16 compared to day 0 in all treatment groups (Fig. 3 and Table 7).

**Table 7:** Joint space width (mm) in different groups on different days

Groups	DAY 0		DAY 7		DAY 16	
	Lateral	Medial	Lateral	Medial	Lateral	Medial
Gr-I	0.82 ± 0.040	0.55 ± 0.022	0.65 ± 0.062	0.40 ± 0.052	0.50 ± 0.058	0.30 ± 0.058
Gr-II	0.83 ± 0.033	0.57 ± 0.021	0.72 ± 0.031	0.45 ± 0.033	0.62 ± 0.03	0.43 ± 0.042
Gr-III	0.85 ± 0.022	0.53 ± 0.021	0.68 ± 0.031	0.38 ± 0.017	0.57 ± 0.033	0.38 ± 0.031
Gr-IV	0.85 ± 0.022	0.55 ± 0.022	0.73 ± 0.033	0.40 ± 0.037	0.63 ± 0.021	0.42 ± 0.021

[*n* = 6]

**Fig. 4:**
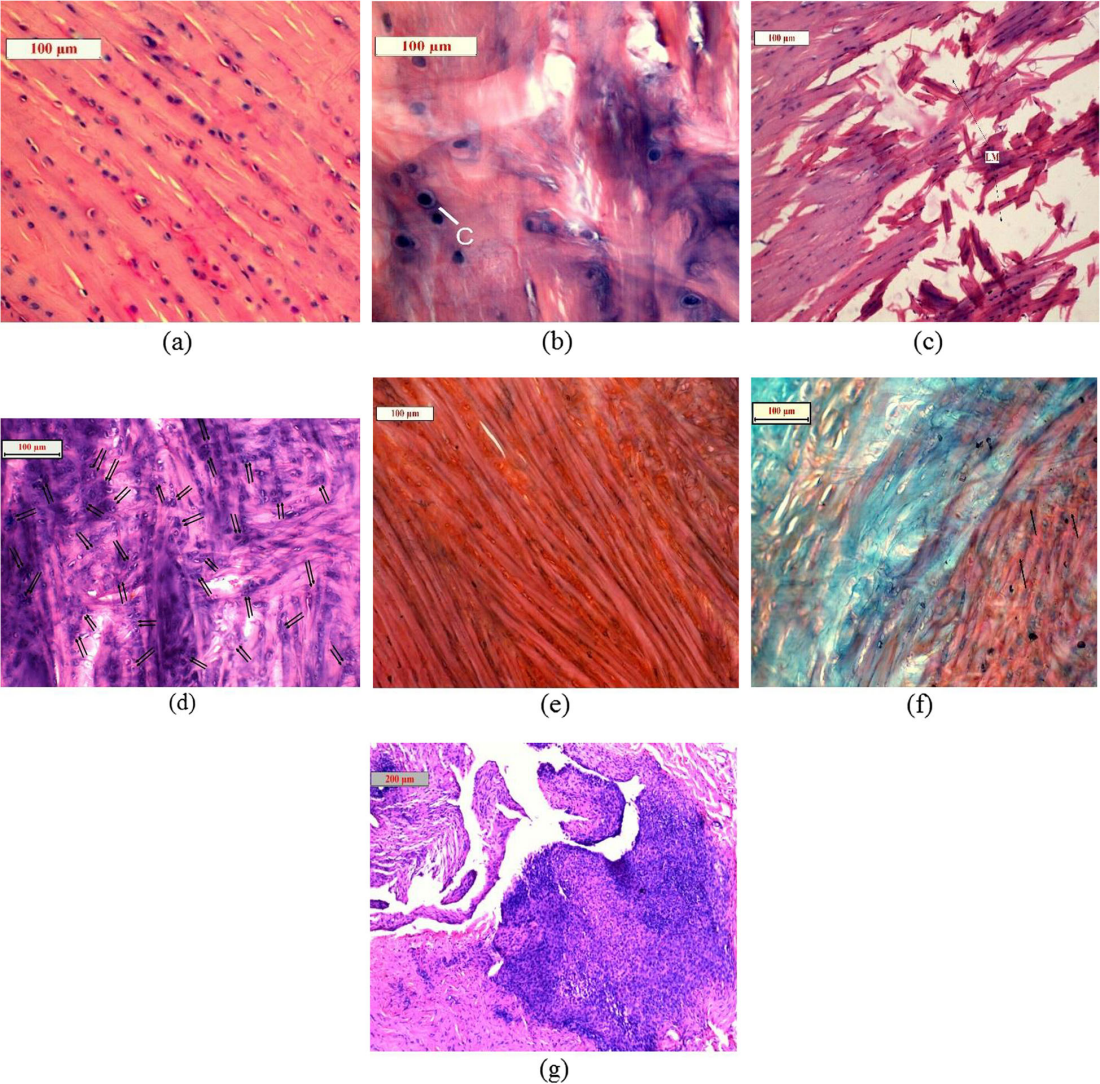
Micrographic view of meniscus of healthy control rabbit showing normal matrix under H and E stain **(a)**; arthritic control rabbit showing very less cellularity under H and E stain **(b)**; arthritis control rabbit showing extensive loss of matrix under H and E stain **(c)**; arthritic control rabbit showing cloning of chondrocytes under H and E stain **(d)**; healthy control rabbit showing high amount of proteoglycan under Safranin O stain **(e)**; arthritic control rabbit showing very low amount of proteoglycan under Safranin O stain **(f)**; arthritic control rabbit showing high amount of pannus under H and E stain **(g)**

An overall histopathological scoring (Table 8) was performed on the basis of 5 parameters. A normal healthy meniscus was scored as 0 for all the parameters. Lesser the score meant better the efficacy. In arthritic control group, the score was found to be highest (14.4) whereas Gr-II possessed the lowest score (7.3). Gr-IV got a score of 8.2 which was very closer to Gr-II score. All the treated groups possessed lower score compared to Gr-I (Fig. 4).

**Table 8:** Overall histomorphological scoring of different groups

Group	Overall Score
Normal healthy	0
Gr-I	14.4
Gr-II	7.3
Gr-III	11.0
Gr-IV	8.2

## Discussion

**Fig. 5:**
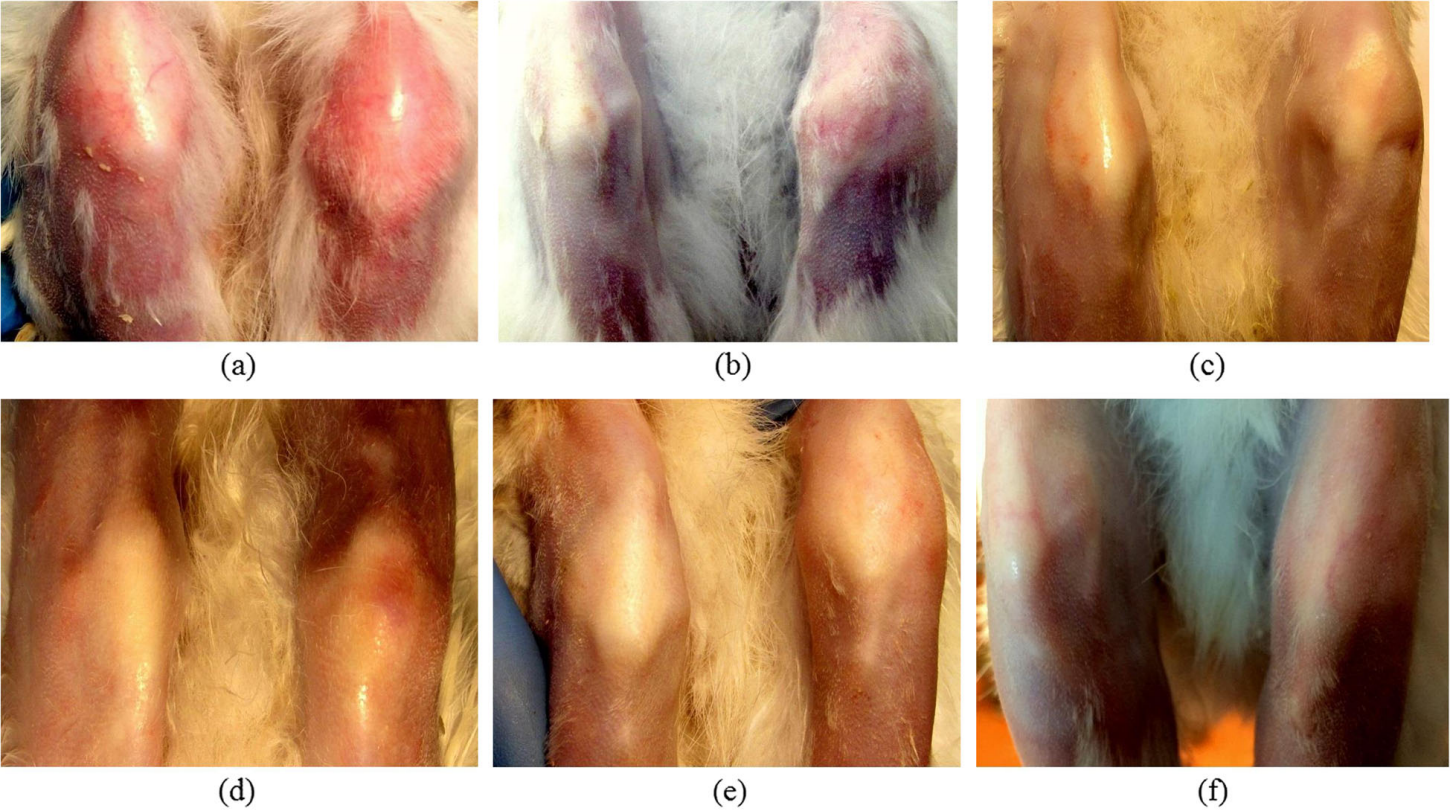
Gross appearance of the left knee showing progression of septic arthritis in arthritic control group on day 7 **(a)** and day 16 **(b)**; HDEETIL treated group on day 7 **(c)** and day 16 **(d)** and linezolid treated group on day 7 **(e)** and day 16 **(f)**

The ethanolic extract of *Tamarindus indica* L. leaves was recorded to have no adverse effect during sub-acute toxicity study. Therefore, the dose rates of 500 and 1000 mg kg^− 1^ were used for efficacy study in induced septic arthritis. Induction of septic arthritis was confirmed by histomorphological, biochemical and microbiological parameters. Culture of the collected pus from affected joint showed typical colonies of *S. aureus* which was further confirmed by PCR. EETIL was found to have a good antimicrobial potential against *S. aureus* that caused septic arthritis. Synovial LDH activity was observed to be elevated more than 1000 U L^− 1^ on day 7 in all the groups. In accordance with the present findings, it was also reported [24] that samples of synovial fluid with proven septic arthritis shows marked elevations in LDH (mean 1279 units). Both the dose rates of EETIL were found to be effective to reduce the synovial LDH level. The HDEETIL was recorded to be more effective than LDEETIL and showed efficacy closer to LNZ. Total protein level in synovial fluid was observed to be above 4.5 g dL^− 1^ on day 4 following inoculation of 10^4^ c.f.u. mL^− 1^ of *S. aureus* in the left stifle joint of rabbits which indicated significant inflammation in the joint of all the experimental groups. The simultaneous ratio of total protein in serum and synovial fluid in EETIL treated groups approached the ratio of LNZ treated group on day 16. In the presence of bacterial infection, synovial fluid glucose may be at least 25 mg dL^− 1^ lower than a simultaneous blood glucose [25]. In our present study, the glucose difference blood and synovial fluid was found to be lower than 25 mg dL^− 1^ on day 16 in Gr-II and Gr-IV (Table 5). Serial digital radiographs of stifle joint at day 0, 7 and 16 were taken and measured digitally. The narrowing of joint space width in untreated control group might be due to aggravation of joint infection. The present findings were similar to the findings of Jacobson et al. (2008) [26]. The narrowing of joint space occurred due to destruction of sub-chondral bone and cartilage on both sides of joint [27]. In septic arthritis, bacterial antigens caused cytokine proliferation [28] inside the joint and activate chondrocyte proteases [29] which in turn caused inflammation of joint. Restriction of joint space narrowing might be due to recovery of joint infection as *Tamarindus indica* L. leaves have antibacterial effect against *S. aureus* including methicillin resistant *S. aureus* [2, 3]. Septic arthritis is a condition of sepsis and inflammation of joint which needs a check of both bacterial multiplication and progression of inflammation at the same time. Phytochemicals present in *Tamarindus indica* L. leaves like saponins, tannins [30, 31] and essential oil especially nerol and linalool may produce potential antimicrobial activity [32]. Potent anti-inflammatory as well as anti-nociceptive actions of the hydroethanolic extract of *Tamarindus indica* L. leaves as reported earlier might be added advantage to exert anti-arthritic effect of the extract in septic arthritis [5]. Induction of septic arthritis produced histomorphological changes like loss of matrix, loss of cellularity, cloning of chondrocytes, adhesion of pannus and loss of proteoglycans in the meniscal cartilage in left stifle joint of rabbits. However, linezolid and HDEETIL caused significant improvement of these histomorphological parameters in arthritis. LDEETIL also showed promising results but are less marked compared to HDEETIL. Treatment with HDEETIL and LNZ significantly reduced the clinical symptoms of septic arthritis like profuse swelling, redness and lameness of the affected left leg in the treated rabbits. Gross appearance of the affected knee in different groups was displayed in Fig. 5 which showed improvements in LNZ and ethanolic extract treated groups.

## Conclusion

The present study suggested that both EETIL and LNZ at the proposed dose rates were effective against septic arthritis caused by *Staphylococcus aureus* in rabbits. Septic arthritis generally requires a long term treatment by antibiotic. Therefore, the ethanolic extract of *Tamarindus indica* L. Leaves at 1000 mg kg^− 1^ orally for consecutive 14 days may be an alternative option for treatment of septic arthritis not only in animals but also in human beings as it showed significant antibacterial activity against the causative bacteria and clinical improvement of septic arthritis.

## Data Availability

The datasets used and/or analysed during the current study are available from the corresponding author on reasonable request.

## Abbreviations

°C: Degree centigrade
ALP: Alkaline phosphatase
ALT: Alanine transaminase
AST: Aspartate aminotransferase
BUN: Blood urea nitrogen
c.f.u.: Colony forming unit
CCl_4_: Carbon tetrachloride
CLSI: Clinical & laboratory standards institute
CPCSEA: Committee for the Purpose of Control and Supervision of Experiments on Animals
dL^− 1^: Per decilitre
EETI: Ethanolic extract of *Tamarindus indica* L
gm: Gram
Gr: Group
HDEETIL: High Dose Ethanolic Extract of *Tamarindus indica* L. leaves
IAEC: Institutional Animal Ethics committee
IBSC: Institutional Biosafety Committee
IL: Interleukin
kg: Kilogram
kg^− 1^: Per kilogram
L: Litre
L.: Carl Linnaeus
L^− 1^: Per litre
LDEETIL: Low Dose Ethanolic Extract of *Tamarindus indica* L. leaves
LDH: Lactate dehydrogenase
LNZ: Linezolid
MBC: Minimum bactericidal concentration
MIC: Minimum inhibitory concentration
min: Minute
MJSW: Medial joint space width
mL: Millilitre
mL^− 1^: Per millilitre
mm: Millimetre
MRSA: Methicillin resistant *Staphylococcus aureus*
OECD: Organisation for Economic Co-operation and Development
PCR: Polymerase chain reaction
PGE_2_: Prostaglandin E_2_
rpm: Revolutions per minute
*S. aureus*: *Staphylococcus aureus*
SGOT: Serum glutamic oxaloacetic transaminase
SGPT: Serum glutamic pyruvic transaminase
TCA: Trichloroacetic acid
U: Unit
v/v: Volume by volume
WBUAFS: West Bengal University of Animal and Fishery Sciences
μg: Microgram
